# Investigation on Surface Integrity of Nodular Cast Iron QT700-2 in Shape Adaptive Grinding

**DOI:** 10.3390/mi14020276

**Published:** 2023-01-20

**Authors:** Liansheng Zhao, Jingjie Zhang, Jin Du, Binxun Li, Jiamao Zhang, Guosheng Su

**Affiliations:** 1 School of Mechanical Engineering, Qilu University of Technology, Shandong Academy of Sciences, Jinan 250300, China; 2 Shandong Institute of Mechanical Design and Research, Jinan 250300, China

**Keywords:** nodular cast iron, shape adaptive grinding (SAG), grinding parameters, surface integrity

## Abstract

Nodular cast iron QT700-2 is extensively used in automobile engine crankshaft parts due to its prime mechanical properties. The journal of a crankshaft is a curved surface, and traditional wheel grinding easily causes grinding burn and surface and subsurface damage. Shape adaptive grinding (SAG) is a flexible grinding technology, which has the advantages of low grinding force and temperature, and good grinding quality. It is suitable for machining curved surface parts with complex shapes. Therefore, the SAG surface integrity of nodular cast iron QT700-2 was experimentally investigated. The influence of grinding parameters on grinding force, material removal rate, grinding temperature, and surface integrity was studied, and the machining performance of SAG tools was evaluated. It was concluded that the grain size in SAG is the most important factor affecting the grinding force, material removal rate, and surface roughness; the influence of SAG grinding is very weak, mainly removing the workpiece material. Then, the influence law of SAG technology on the surface integrity of nodular cast iron QT700-2 was summarized, and the optimal grinding parameters were obtained, providing a reference for the curved surface grinding of nodular cast iron QT700-2 in the future.

## 1. Introduction

Nodular cast iron QT700-2 has good casting processability and excellent strength and toughness. It is often used to make automobile engine crankshaft parts [1,2,3]. The journal of a crankshaft is a curved surface, which is very difficult to fine machine by traditional wheel grinding. When grinding a crankshaft, the touch between the hard grinding wheel and the crankshaft produces a strong interaction, resulting in grinding burn, surface and subsurface damage on the machined surface, and the machined quality being low [4,5]. Therefore, polishing is required to reduce the surface roughness and make it suitable for final use. Grinding is the most ideal fine machining method for nodular ductile iron QT700-2. However, a rigid grinding apparatus makes it difficult to achieve nanoscale polishing of nodular ductile iron QT700-2.

Shape adaptive grinding (SAG) is a comparatively new fine machining technology, which can fine machine hard and brittle materials to obtain nanometer surface roughness [6,7,8,9]. This is a flexible polishing technology. The main body uses an elastic rubber sacculus (the interior is filled with constant pressure gas, and the air pressure is used as the grinding force), and hard grains are attached to the surface. During the grinding process, the elastic sacculus is deformed by force to fit the workpiece’s curved surface, and grains on the surface are squeezed to remove the workpiece material [10,11,12].

Zeng et al. studied the effect of grinding parameters on material removal rate through a SAG device, and found that MRR nonlinearly increases with the increase in the feed angle, linearly increases with the increase in the spindle speed, and increases at first and then reduces with the increase in the tool compression [13]. Beaucamp et al. used a SAG tool to grind CVD silicon carbide materials [14,15], measured grinding forces under various grinding parameters, compared them with the observed grinding patterns, and studied the process mechanism of SAG [16]. By grinding with grains of different sizes (3, 9, 40 μm) and observing the grinding surface, it was found that the air pressure had no effect on SAG and that grain size was the main factor for the transition from fracture to ductile mode. Finally, the machined surface roughness was lower than 0.4 nm and the material removal rate was as high as 100 mm^3^/min [17,18]. However, because the internal gas of the SAG tool needs to maintain a constant pressure, it can only be used in a specific experimental device, which limits its application.

Ghosh et al. improved the SAG technology by changing the elastic balloon used by the SAG tool into a solid and replacing air pressure with elastic force as the grinding force [19]. A variety of SAG tools have been proposed, and the effects of grinding parameters on normal force, material removal rate, and surface roughness have been studied. It was found that the increase in the tool compression could effectively increase MRR and reduce surface roughness and that the feed rate had little effect on the normal force [20]. A horizontal SAG device was designed by imitating the grinding wheel for grinding experiments, and it was found that due to the existence of elastic materials, the number of active grains in SAG was 30% higher than that in the grinding wheel and that grain wear was the main wear mechanism [21]. However, the solid elastic grinding apparatus has a large mass and is greatly affected by centrifugal force [22]. In addition, the improved SAG tool is a plane grinding apparatus, which is not suitable for grinding curved parts.

Based on the above research, a spherical SAG tool was proposed, which is suitable for surface finishing, and a range of grinding experiments were designed. The influence of grinding parameters on grinding force, material removal rate, surface roughness, grinding temperature, work hardening, and microstructure was studied, and the machining performance of SAG tools was evaluated, providing a reference for the surface grinding of nodular cast iron QT700-2 material in the future.

## 2. Experiment

### 2.1. Workpiece Material

**Figure 1:**
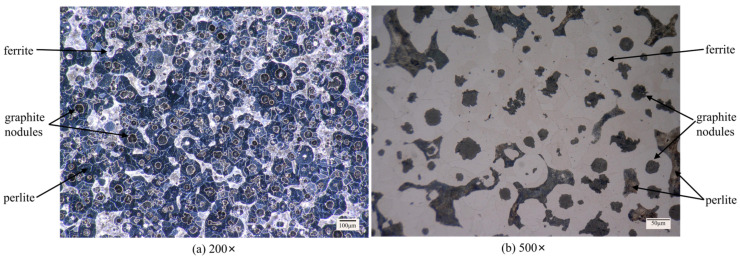
Microstructure of nodular cast iron QT700-2: (**a**) 200×, (**b**) 500×.

Nodular cast iron QT700-2 was used as the workpiece material under in grinding experiments. The metallographic sample was made from an unground workpiece. After polishing and corrosion, the element content was measured by EDX, and then the sample surface was observed with an optical microscope. The chemical components of QT700-2 are listed in Table 1. Figure 1 shows an optical image of the microstructure of nodular cast iron QT700-2. It can be seen that the matrix tissue of the sample was composed of ferrite, pearlite, and spherical graphite [23].

The workpiece blank specification of nodular cast iron QT700-2 was 70 mm × 70 mm × 80 mm, and the sample was prepared by face milling. After milling, the workpiece surface roughness was Ra ∈ [1.45,1.55] μm.

### 2.2. Experimental Setup

**Figure 2:**
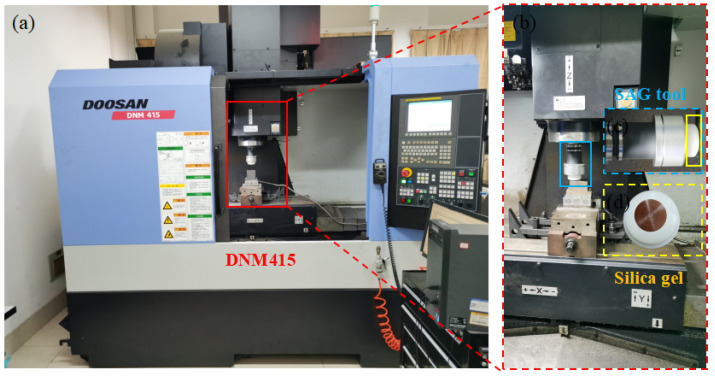
SAG experimental setup: (**a**) DNM415 CNC Machining Center, (**b**) SAG tool composition.

The grinding experiment was conducted on a three-axis NC milling center (DNM415, Doosan Machine Tool (China) Co., Ltd., Yantai, China) (as shown in Figure 2). The SAG tool was connected to a cutter shank and installed on a machine spindle. The SAG tool structure was as follows: (1) the upper layer was an aluminum alloy (7075-T651) shell (φ 84 mm), (2) the middle layer was an elastic material (Shore 30 silica gel), and (3) the lower layer was a diamond polishing sheet (φ 29 mm).

### 2.3. Experimental Method

For the experiment, we adopted the single factor experimental method. A total of 16 groups of trials were carried out. The effects of grinding parameters (grain size (P), spindle speed (N), feed rate (Vf), and tool compression (W)) on grinding force, material removal rate, surface roughness, grinding temperature, work hardening, and microstructure were studied. SAG process parameters are shown in Table 2. In an effort to ensure the dependability of the experimental results, each group of experiments was reduplicated 3 times to obtain an average value.

### 2.4. Experimental Data Measurement

The grinding force was measured online with a cutting dynamometer (Kistler 9129AA, Kistler Instrumente AG, Wintertour, Switzerland). Meanwhile, the grinding temperature was measured with a digital display type K thermocouple and a thermal imager (Flir A315, Shanghai Jianling Electronic Technology Co., Ltd., Shanghai, China). After the grinding experiment, the grinding depth was measured by profilometry (Bruker SPR2000, Slate Intelligent Technology Co., Ltd., Shenzhen, China). The workpiece surface roughness (Ra) was measured with a white light interferometer (Contour Elite K, Brook Scientific Instruments, Madison, WI, USA), and the average value was measured 6 times at random. For the preparation of the metallographic samples, we used WEDM (ZJK 7532A, Suzhou Sanguang Wire Cutting Machine Tool Factory, Suzhou, China). After polishing and etching, the surface microstructure of the sample was observed with a scanning electron microscope (Phenom Prox, Funa Scientific Instrument Co., Ltd., Shanghai, China). The surface microhardness of the sample was measured with a Vickers microhardness tester (HXD-1000TMC, Wuxi Metus Precision Technology Co., Ltd., Wuxi, China), and the load was 200 g. In addition, the workpiece surface morphology was observed with an ultra-deep field 3D viewing microscope (Keyence VHX-5000, Kearns (China) Co., Ltd., Shanghai, China).

## 3. Results and Discussion

### 3.1. Grinding Force

**Figure 3:**
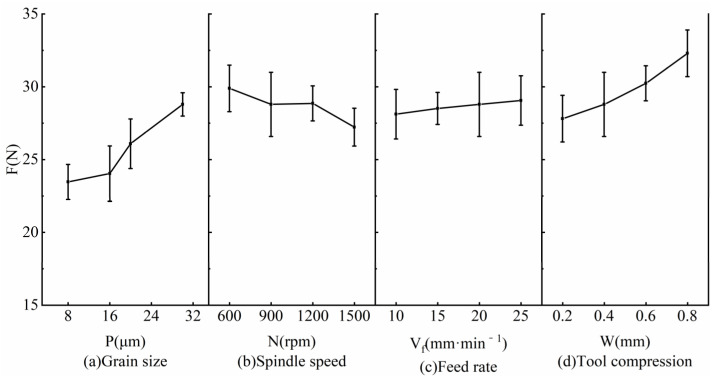
Influence of grinding parameters on grinding force: (**a**) Grain size, (**b**) Spindle speed, (**c**) Feed rate, (**d**) Tool compression.

In an effort to study the influence of grinding parameters on grinding force, grinding experiments were conducted according to the parameters in Table 2, and the grinding force was measured. Each experiment was repeated three times to calculate the average value and standard deviation. The ratio of the standard deviation to the average value of the grinding force (F) was usually less than 7.9%, and the measurement results were valid. Figure 3 shows the influence of the grinding parameters on the grinding force. From here, we see that with the increase in the grain size from 8 μm to 30 μm, F increased by 22.7%. The mechanism is as follows: with the increase in the grain size, the cutting depth of single grain increases and the grinding force increases. With the increase in the spindle speed, F reduced by 8.9%. The reason is that with the increase in the spindle speed, the number of grains per unit area per unit time increases, the cutting depth of the single grain reduces, and F reduces. With the increase in the feed speed of the SAG tool, F increased by 3.3%. It is noted that with the increase in the tool compression, F increased by 16.1%, which was caused by the deformation of the flexible tool.

Based on the above analysis, the grain size has the most remarkable influence on the grinding force, then the tool compression, and then the spindle speed; the feed rate has little influence on the grinding force. For the SAG of nodular cast iron QT700-2, the grinding force is between 23 and 33 N, which is much less than that of traditional nodular ductile iron grinding.

### 3.2. Material Removal Rate

The material removal rate refers to the material mass or volume thrown from the matrix material in a unit of time, which can be calculated by the volume change in the workpiece before and after grinding [24]. The grinding depth was measured by profilometry, which was used to calculate the material removal rate. The material removal volume can be obtained by multiplying the grinding depth by the grinding area. The ratio of the material removal volume to the grinding time is the material removal rate.

**Figure 4:**
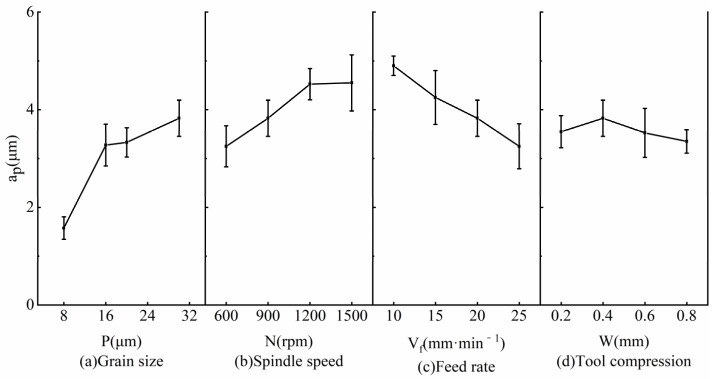
Influence of grinding parameters on grinding depth: (**a**) Grain size, (**b**) Spindle speed, (**c**) Feed rate, (**d**) Tool compression.

Different from traditional rigid wheel grinding, the real grinding depth of SAG is far less than the parameters set by it. Figure 4 shows the influence of grinding parameters on grinding depth. The ratio of the standard deviation to the average of the grinding depth was less than 14.6%, and the measurement result as valid.

The influence of grain size on grinding depth is shown in Figure 4a. With the increase in the grain size from 8 μm to 20 μm, the grinding depth increased from 1.575 μm to 3.825 μm. The mechanism is as follows: with the increase in the grain size, the quantity of grain per unit area reduces, the grinding force on a single grain increases, and the grinding depth increases [20]. Conversely, with the increase in the spindle speed, the grinding depth showed an upward tendency. When the spindle speed increased from 600 rpm to 1500 rpm, the grinding depth increased from 3.25 μm to 4.55 μm, but the increase gradually slowed, as shown in Figure 4b. Its main mechanism can be generalized as follows: (1) With the increase in the spindle speed, the number of grains per unit area per unit time increases, and the grinding depth on a single grain reduces, but the total grinding depth increases [14]. (2) The increase in the spindle speed causes increases in the centrifugal force and the degree of deformation of the flexible tool, the diminution of the total grinding force, and the increase in grinding depth reduces [22]. Figure 4c shows the effect of feed rate on grinding depth. With the increase in the feed rate, the grinding depth showed a significant downward trend. The reason is that with the increase in the feed rate, the number of grains per unit area per unit time reduces, and the total grinding depth reduces. In addition, with the increase in the tool compression, when the tool compression was lower than 0.4 mm, the grinding depth slightly increased. With the further increase in tool compression, the grinding depth slightly reduced. Its main mechanism can be generalized as follows: (1) With the increase in the tool compression, the grinding force increases from 27.81 N to 32.3 N, the grinding area increases from 153.86 mm^2^ to 314 mm^2^, the variation range of the grinding force is less than that of the grinding area, the grinding force of a single grain gradually reduces, and the grinding depth reduces gradually. (2) Due to the excessive material removal and the excessive accumulation of grinding chips when the tool compresses at 0.2 mm, it hinders the next step of material removal, and the grinding depth becomes very shallow.

According to the above analysis, under the current grinding parameters, the influence of various elements on the grinding depth are the feed rate, grain size, spindle speed, and tool compression.

**Figure 5:**
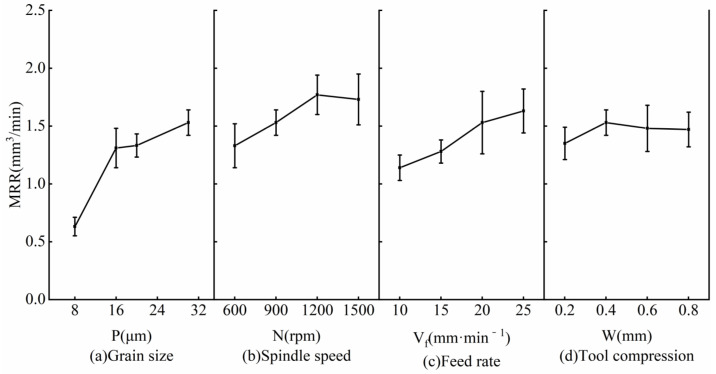
Influence of grinding parameters on material removal rate: (**a**) Grain size, (**b**) Spindle speed, (**c**) Feed rate, (**d**) Tool compression.

The material removal rate is equivalent to the product of the grinding depth and the grinding area per unit of time. The ratio of the standard deviation to the average value of the material removal rate was less than 17.6%, indicating the measurement result is effective. Figure 5 shows the effect of grinding parameters on grinding depth, and the analysis of the results is as follows:

The influence of grain size on the material removal rate is shown in Figure 5a. It can be seen that when the grain size increased from 8 μm to 20 μm, the material removal rate increased from 0.63 mm^3^/min to 1.53 mm^3^/min, and the rising trend was exactly the same as the grinding depth. The mechanism is as follows: with the increase in the grain size, the grinding depth increases, the grinding area remains unchanged, and the material removal rate increases.

Additionally, with the increase in the spindle speed, when the spindle speed was lower than 1200 rpm, the material removal rate slightly increased. With the further increase in the spindle speed, the material removal rate slightly reduced, as shown in Figure 5b. Its main mechanism can be summarized as follows: with the increase in the spindle speed, the centrifugal deformation of the flexible tool increases, the grinding area reduces, and the grinding depth increases. The variation range of the grinding area is greater than the grinding depth, and the material removal rate increases at first and then gradually reduces.

It can be seen from Figure 5c that the material removal rate gradually increased with the increase in the feed rate. When the feed rate increased from 10 mm^3^/min to 25 mm^3^/min, the material removal rate increased from 1.14 mm^3^/min to 1.61 mm^3^/min. The main reason is that with the increase in the feed rate, the grinding area greatly increases, and the grinding depth reduces. The variation range of the grinding area is much greater than the grinding depth, and the material removal rate increases.

Figure 5d shows the influence of tool compression on the material removal rate. With the increase in the tool compression, when it was lower than 0.4 mm, the material removal rate slightly increased. With further increases in the tool compression, the material removal rate slightly reduced. This is because with the increase in the tool compression, the grinding area increases, and the grinding depth first increases and then reduces. The variation range of the grinding area is much smaller than the grinding depth, and the material removal rate first increases and then reduces.

To sum up, under the current grinding parameters, the influence of various factors on material removal rate are grain size, feed rate, spindle speed, and tool compression.

### 3.3. Surface Roughness

Surface roughness is the significant factor to consider when evaluating the quality of a grinding surface. The surface roughness was measured with a white light interferometer, each workpiece surface was measured randomly six times, and the average value and standard deviation were calculated.

**Figure 6:**
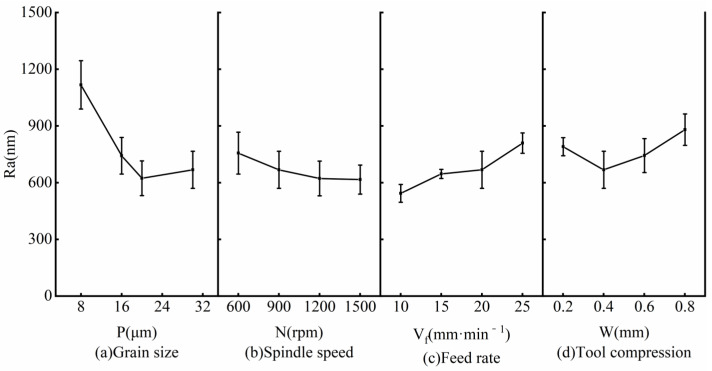
Influence of grinding parameters on surface roughness: (**a**) Grain size, (**b**) Spindle speed, (**c**) Feed rate, (**d**) Tool compression.

Figure 6 shows the influence of the grinding parameters on surface roughness (Ra). The ratio of the standard deviation to the average value of Ra was less than 15%, and the measurement results are valid. As can be seen, Ra varied from 0.55 μm to 1.2 μm.

Additionally, when the grain size was less than 20 μm, Ra reduced with the increase in the grain size, as shown in Figure 6a. After that, Ra increased slightly with the increase in the grain size. The mechanism is summarized as follows: (1) When the grain size is less than 20 μm, the grinding depth increases with the increase in the grain size, the milling traces on the workpiece surface are continuously removed, and Ra reduces. (2) When the grain size is larger than 20 μm, most of the milling traces on the workpiece surface are removed; the larger the size of the grain, the more obvious the grinding trace, and Ra increases. As shown in Figure 6b, with the increase in the spindle speed, Ra gradually reduced, and the decreasing range gradually slowed. When the feed rate increased, Ra rapidly increased, as shown in Figure 6c. Figure 6d shows the influence of tool compression on Ra. With the increase in the tool compression, when the tool compression was lower than 0.4 mm, Ra slightly reduced. With the further increase in tool compression, the grinding depth slightly increased. This is because with the increase in the tool compression, the grinding depth first increases and then reduces, and Ra first increases and then reduces.

According to the above analysis, under the current grinding parameters, the influence of various factors on Ra are grain size, feed rate, tool compression, and spindle speed.

In the finishing of ductile materials (nodular cast iron QT700-2), the accumulation of grinding chips becomes an important influencing factor. As shown in Figure 6a, Ra linearly increased when the grain size was reduced from 20 μm to 8 μm. Among the reasons for this, the difficulty of debris discharge caused by the reduction of the wear particle size is an important one.

In addition, during dry grinding, over time, grinding chips continue to accumulate on the machined surface and affect the removal of the workpiece materials by the grinding apparatus. Spraying cutting fluid on the grinding area during grinding can control heat generation, reduce oxidation, and help to remove debris from the grinding surface, reducing debris accumulation.

According to the experiment results, the optimal grinding parameters (grain size 20 μm, spindle speed 1500 rpm, feed rate 10 mm/min, and tool compression 0.4 mm) were selected.

**Figure 7:**
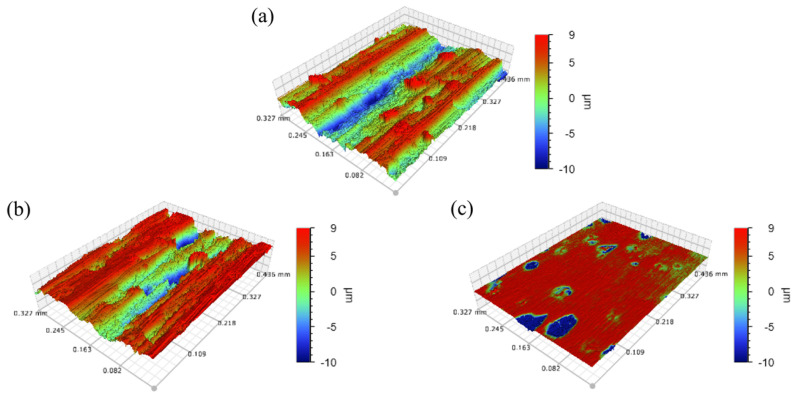
The 3D topography of the grinding surface: (**a**) Before grinding (Ra = 1.541 µm), (**b**) Dry grinding after 16 passes (Ra = 0.952 µm), (**c**) Wet grinding after 16 passes (Ra = 0.19 µm).

The experiment was carried out 16 times in a row. Each time, grinding lasted 4 min, the change in the 3D morphology of the workpiece surface was observed, and Ra was measured. Figure 7a shows the 3D morphology of the workpiece surface before grinding, and the groove left by milling can be seen. Figure 7b,c show the surface morphology after 16 rounds of dry and wet grinding. Compared with wet grinding, obvious residual milling traces were still observed on the dry grinding surface. The Ra values of dry and wet grinding surfaces were 0.952 μm and 0.19 μm, respectively.

**Figure 8:**
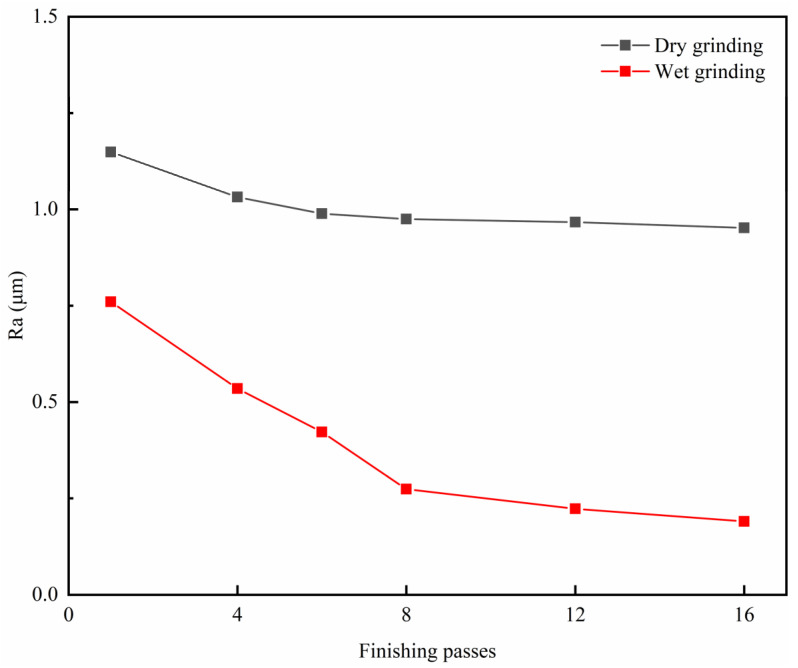
Variation in surface roughness with the finishing passes for both dry and wet grinding.

**Figure 9:**
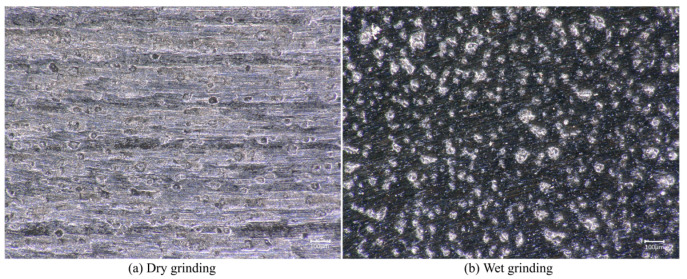
The optical image of the grinding surface (200×): (**a**) Workpiece surface after dry grinding, (**b**) Workpiece surface after wet grinding.

Figure 8 shows the range in the Ra of dry and wet grinding with grinding duration. It was observed that Ra reduced with the increase in the grinding duration. For dry grinding, the change in Ra was very small. It can be seen that the milling traces on the surface of the workpiece were obvious, and a lot of debris remained, as shown in Figure 9a. For wet grinding, the variation in Ra was much larger than that of dry grinding. On the basis of Figure 9b, most of the milling traces on the workpiece surface disappeared.

To sum up, the use of cutting fluid can obviously improve the grinding quality. Using this method, an Ra 0.19 μm was realized in 128 min.

### 3.4. Grinding Temperature

According to Figure 6, the experimental parameters corresponding to the maximum material removal rate (grain size 20 μm, spindle speed 900 rpm, feed rate 20 mm/min, and tool compression 0.4 mm) were selected, the grinding experiment was conducted, and then the grinding temperature was measured.

**Figure 10:**
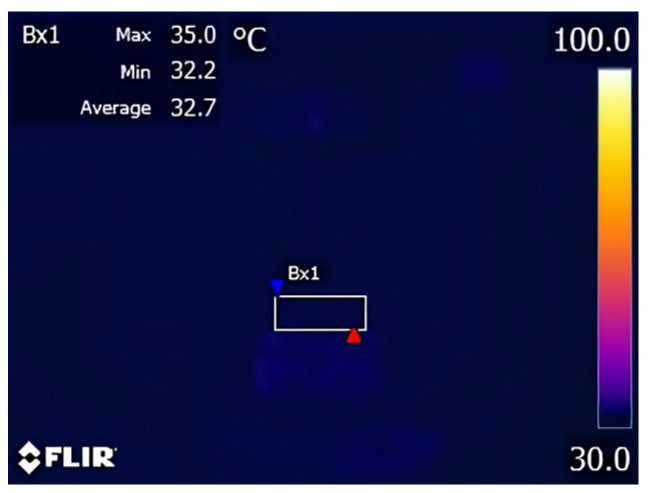
Infrared image during grinding.

An infrared thermometer and a digital explicit K-type thermocouple were used for comparative measurement during grinding. In the grinding experiment, the infrared thermal imager did not capture the obvious temperature difference of the grinding surface, as shown in Figure 10, which indicated that the heat generated by the removal of the SAG material was too small and did not cause a large temperature change on the grinding surface.

**Figure 11:**
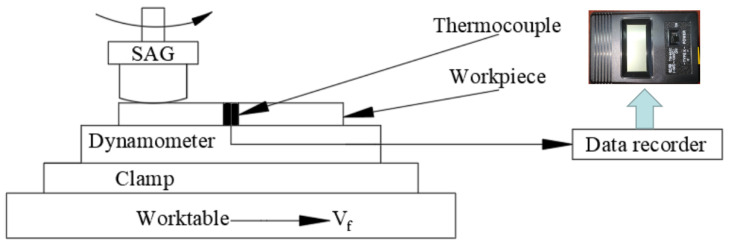
Temperature measurement diagram of K thermocouple.

The diagram of the temperature measurement experiment with a digital explicit K thermocouple [25] is shown in Figure 11. A slot (0.5 mm × 2 mm × 35 mm) was cut perpendicular to the workpiece surface, embedded the thermocouple, and fixed with glue. In three repeated experiments, the grinding surface temperature increased from 25.8 °C to 43.7 °C, which was confirmed by the results of the infrared thermometer.

### 3.5. Work Hardening

During grinding, the surface material of the workpiece is affected by both the grinding temperature and the grinding force, and the phenomena of thermal softening and work hardening occur. Thermal softening and work hardening have different effects on the microhardness of workpiece sections [26,27].

For the SAG tool, the lower grinding force (up to 33 N) did not generate large cutting forces, and the extremely low grinding temperature (up to 43.7 °C) did not cause thermal softening of the machined surface, so the machining influence layer was very small.

**Figure 12:**
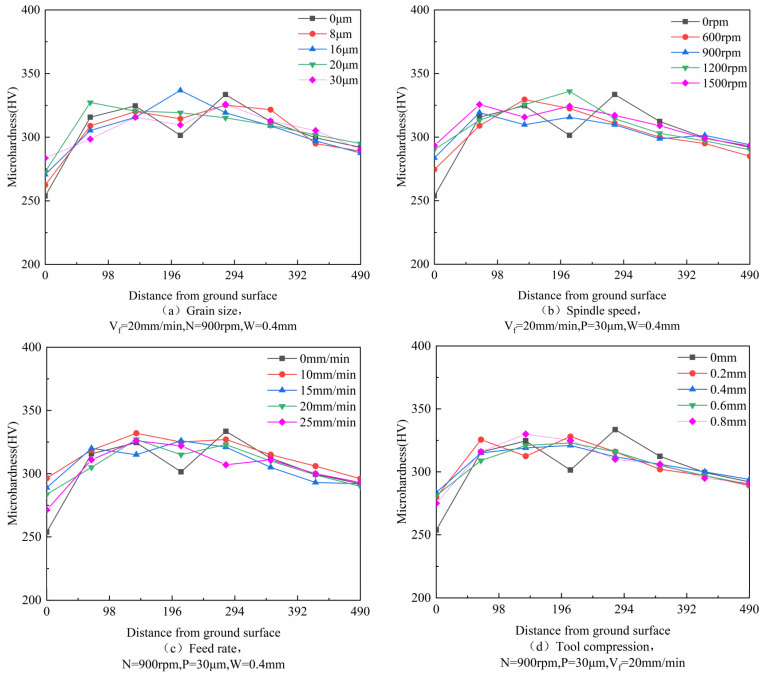
Microhardness depth distribution of sample surface under different grinding conditions: (**a**) Grain size, (**b**) Spindle speed, (**c**) Feed rate, (**d**) Tool compression.

The matrix hardness of nodular cast iron QT700-2 was 292 HV (no heat treatment). Figure 12 shows the microhardness depth distribution of the sample surface under different grinding parameters. It can be found that all the microhardness results had similar profiles and that the depth of the hardened layer was basically the same. With the increase in the distance from the grinding surface, the microhardness increased at first and then reduced to the matrix hardness. The trend of the change in microhardness was caused by the milling process.

**Figure 13:**
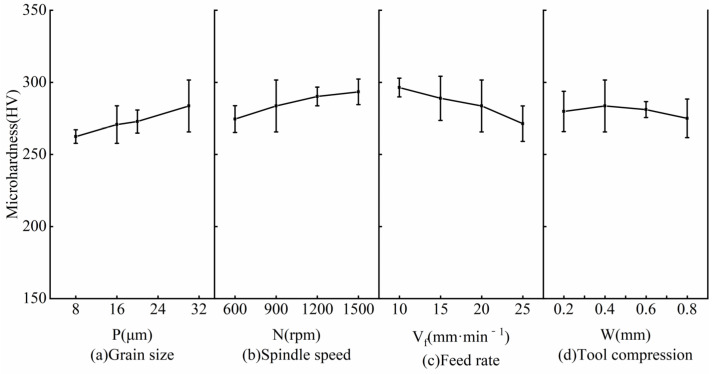
Influence of grinding parameters on surface microhardness: (**a**) Grain size, (**b**) Spindle speed, (**c**) Feed rate, (**d**) Tool compression.

Figure 13 shows the influence of different process parameters on the surface microhardness of the workpiece. As can be seen, the influence trend was basically the same as the grinding depth trend. The specific reasons are as follows: there is residual tensile stress on the workpiece surface after milling, and the removal of the workpiece material by the SAG releases the residual tensile stress. The greater the grinding depth, the higher the microhardness.

### 3.6. Microstructure

According to the data in Figure 6, the workpiece with the smallest surface roughness was selected to make the metallographic sample and compared with the metallographic sample of the unground workpiece. The change in the sample surface was observed.

**Figure 14:**
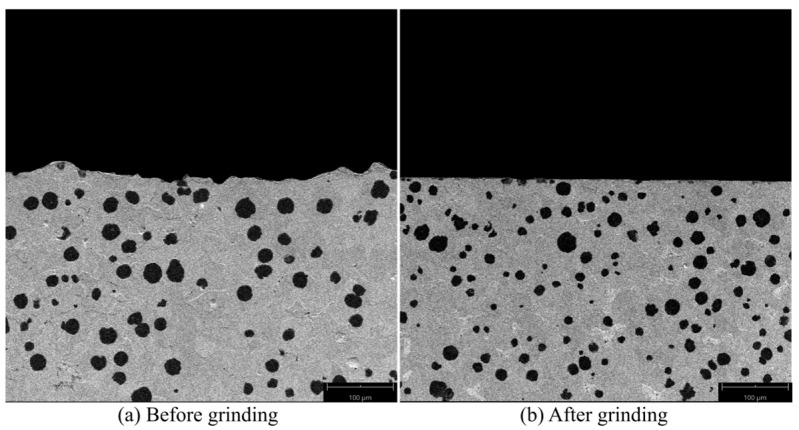
SEM image of sample surface (500×): (**a**) Sample surface before grinding, (**b**) Sample surface after grinding.

Figure 14 is the SEM diagram of the sample surface. It can be seen that the workpiece surface before grinding was not smooth and there were defects such as grooves and pits, which would affect the service life of the workpiece. After grinding, the surface of the workpiece became smooth and flat, and most of the surface flaw was removed.

The grain size of nodular cast iron QT700-2 is more than 40 μm, but the single grinding depth in the experiment was less than 4.9 μm, which is much smaller than that of the nodular cast iron. Therefore, the tissue fibrosis deformation caused by shear tension did not occur, and the processing influence layer was shallow. At the same time, due to the low temperature in the experiment, there was no thermal damage, which was beneficial for improving the surface quality of the workpiece.

## 4. Conclusions

In an effort to solve the problems with finishing a curved surface, a SAG tool was proposed. In order to clarify the rule of the SAG surface integrity of nodular cast iron QT700-2, a series of experiments was conducted, and the main conclusions are as follows:

- A spherical SAG tool can be designed to grind arbitrary curved surfaces larger than the curvature of the tool.
- The grain size in SAG is the main factor affecting the grinding force, material removal rate, and machined surface roughness. The use of cutting fluid can effectively reduce the impact of debris accumulation.
- The grinding force (up to 33 N) and grinding temperature (up to 43.7 °C) of the SAG tool are very low, and the machining influence layer is very small. During grinding, the workpiece material can be mostly removed.
- The recommended parameters of grinding include a grain size of 20 μm, spindle speed of 1500 rpm, feed rate of 10 mm/min, and tool compression of 0.4 mm. After continuous grinding for a period of time, the workpiece surface roughness decreases from Ra 1.541 μm to Ra 0.19 μm, which is lower than the Ra 0.2 μm finishing roughness requirement of the crankshaft.

## Figures and Tables

**Table 1:** The chemistry of nodular cast iron QT00-2 (mass fraction, %).

C	Si	Mn	S	P	Fe
24.57	2.17	0.16	0.021	0.047	Bal.

**Table 2:** Parameters in shape adaptive grinding experiments.

Grains	Diamond
Grain size	8, 16, 20, 30 μm
Spindle speed	600, 900, 1200, 1500 rpm
Feed rate	10, 15, 20, 25 mm/min
Tool compression	0.2, 0.4, 0.6, 0.8 mm

## Data Availability

The data supporting this study’s findings are available from the corresponding author upon reasonable request.
